# Gambierone and Sodium Channel Specific Bioactivity Are Associated with the Extracellular Metabolite Pool of the Marine Dinoflagellate *Coolia palmyrensis*

**DOI:** 10.3390/md21040244

**Published:** 2023-04-15

**Authors:** Alexander K. Leynse, Elizabeth M. Mudge, Andrew D. Turner, Benjamin H. Maskrey, Alison Robertson

**Affiliations:** 1 School of Marine & Environmental Sciences, University of South Alabama, 600 Clinic Drive, Mobile, AL 36688, USA; 2 Dauphin Island Sea Lab, 101 Bienville Boulevard, Dauphin Island, AL 36528, USA; 3 National Research Council of Canada, 1411 Oxford Street, Halifax, NS B3M3Z1, Canada; 4 Center for the Environment, Fisheries, and Aquaculture Science, Barrack Road, Weymouth DT4 8UB, UK

**Keywords:** phycotoxin, benthic, marine biotoxin, ecology, ciguatera, harmful algal bloom, sodium channel

## Abstract

Tropical epibenthic dinoflagellate communities produce a plethora of bioactive secondary metabolites, including the toxins ciguatoxins (CTXs) and potentially gambierones, that can contaminate fishes, leading to ciguatera poisoning (CP) when consumed by humans. Many studies have assessed the cellular toxicity of causative dinoflagellate species to better understand the dynamics of CP outbreaks. However, few studies have explored extracellular toxin pools which may also enter the food web, including through alternative and unanticipated routes of exposure. Additionally, the extracellular exhibition of toxins would suggest an ecological function and may prove important to the ecology of the CP-associated dinoflagellate species. In this study, semi-purified extracts obtained from the media of a *Coolia palmyrensis* strain (DISL57) isolated from the U.S. Virgin Islands were assessed for bioactivity via a sodium channel specific mouse neuroblastoma cell viability assay and associated metabolites evaluated by targeted and non-targeted liquid chromatography tandem and high-resolution mass spectrometry. We found that extracts of *C. palmyrensis* media exhibit both veratrine enhancing bioactivity and non-specific bioactivity. LC-HR-MS analysis of the same extract fractions identified gambierone and multiple undescribed peaks with mass spectral characteristics suggestive of structural similarities to polyether compounds. These findings implicate *C. palmyrensis* as a potential contributor to CP and highlight extracellular toxin pools as a potentially significant source of toxins that may enter the food web through multiple exposure pathways.

## 1. Introduction

Tropical epiphytic dinoflagellate populations produce complex profiles of bioactive secondary metabolites, some of which can have harmful effects towards humans and marine organisms. A primary concern is ciguatera poisoning (CP), which is regarded as the most problematic seafood poisoning caused by harmful algal blooms (HABs [1,2]). A suite of structurally related metabolites, called ciguatoxins (CTXs), are recognized as the primary causative agents of CP and appear to be exclusively produced by the genera *Gambierdiscus* and *Fukuyoa* [3]. Fish and other herbivorous animals become contaminated with CTXs as they inadvertently consume toxin-producing dinoflagellate cells, while grazing on associated benthic macrophytes. The toxins appear to bioaccumulate in herbivores and are transferred to higher trophic levels that are commonly harvested for human consumption [1,2]. Many of the ecological and physiological processes that ultimately lead to CP outbreaks remain poorly understood. For example, fish toxicity rarely correlates with the abundance of *Gambierdiscus* cells [4,5,6]. Furthermore, the occurrence of toxic fish within a given region can vary spatially and temporally [7], while the symptoms experienced by victims of CP are often variable [8,9]. The often reported “enigmatic nature” of CP outbreaks may be attributed to the complexity of benthic epiphyte communities referred to by some as a holobiont [10,11,12,13]. This macroalgal holobiont is regarded as a complex ecological unit composed of highly specialized symbiotic interactions (i.e., nutrient cycling and chemical interactions via secondary metabolites), important not only to the host but all organisms involved [13,14]. Certainly, biotic and chemical interactions occurring within the holobiont may play a role in community structure and the proliferation of benthic HAB species [13]. Although *Gambierdiscus* and *Fukuyoa* are the only known producers of CTXs to date, other frequently cohabiting genera also produce bioactive secondary metabolites, e.g., okadaic acid (OA) and maitotoxins (MTXs), and, therefore, may further complicate the toxin profiles associated with CP. 

The dinoflagellate genus *Coolia* commonly occurs among tropical epiphyte communities and has been shown to exhibit significant chemical diversity and associated bioactivity. As such, *Coolia* spp. have been implicated as contributors to CP. The genus consists of eight genetically distinct species: *C. monotis*, *C. malayensis*, *C. tropicalis*, *C. canariensis*, *C. palmyrensis*, *C. santacroce*, *C. guanchica*, and *C. areolata* [15]. The most broadly distributed species is *C. malayensis*, which has been found in every ocean and in both tropical and temperate waters [16,17]. The remaining species appear to be restricted to tropical waters [18,19,20,21]; however, limited reporting likely confounds the species’ distributions across regions [16,19].

Numerous studies have incorporated bioassays and liquid chromatography tandem mass spectrometry (LC-MS/MS) or high-resolution mass spectrometry (HR-MS) to explore cell extracts from *Coolia* spp. for bioactivity and known or novel toxins [22,23,24,25]. The first identification of a bioactive secondary metabolite produced by *Coolia* spp. was reported by Holmes and colleagues [23]. In their study, extracts from *C. tropicalis* cells (previously misidentified as *C. monotis*) were partitioned to yield hexane, butanol, and water-soluble fractions. The butanol soluble fraction was found to be toxic (LD_50_ = 1 mg/kg) when tested on the mouse bioassay (MBA), indicating that the observed response is caused by predominantly lipophilic compounds. In the same extracts, a mono-sulphated analog of yessotoxin (YTX) (*m*/*z* = 1062) was identified via LC-MS/MS and named cooliatoxin [23]. However, structural elucidation of cooliatoxin was never reported, and no other studies have confirmed the presence of cooliatoxin in *Coolia* extracts. Cell extracts of a *C. malayensis* strain from Hong Kong were also reported to contain novel sulfated polycyclic ether structures that may represent undescribed yessotoxin analogs, but no toxicity data were presented [22]. Multiple strains of *C. malayensis* have been reported to produce the polycyclic ether toxins gambierone and 44-methylgambierone, while some strains of *C. tropicalis* produce 44-methylgambierone [25,26]. Numerous studies have reported that cell extracts from *Coolia* spp. exhibit toxicity to a variety of larval organisms, including *Artemia* spp. [27,28,29,30]; the sea snail *Haliotis virginea* [27]; sea urchin *Heliocidaris crassispina* [28]; and marine medaka *Oryzias melastigma* [31]. Furthermore, toxicity to several cell lines, including Rhabdomycarsoma cells and sheep erythrocytes, has been reported [20,32]. However, none of these experimental designs considered relevant exposure pathways or doses. 

While other studies examined the intracellular toxin quotas of benthic HAB genera (including *Coolia*) or toxicity associated with dinoflagellate mucus [33,34,35], few have evaluated the bioactive secondary metabolites that occur in media [36]. While culture studies may not accurately reflect the natural environment, the release of toxins from cultured cells may provide an insight into their biological roles, for instance, their potential use in grazing deterrence, quorum sensing (e.g., to manage/manipulate population density), or as allelochemicals (e.g., to reduce fitness of competitors). Furthermore, dissolved toxin pools may enter the food web through alternative exposure pathways (i.e., gills) and contribute to CP. In this study, we used the sodium channel specific mouse neuroblastoma (N2a-MTT) assay and LC-HR-MS to investigate the chemical diversity and bioactivity associated with synthetic absorbent resin (HP-20) captured media extracts from a *C. palmyrensis* culture. Interestingly, a semi-purified hydrosoluble fraction from our extracts exhibited both non-specific and CTX–like bioactivity and was found to contain gambierone and a variety of additional novel compounds with possible polycyclic ether backbones. These results further implicate this species as a contributor to the chemical diversity of algal sources in CP regions and highlights the relevance of extracellular toxin pools in ecosystems and human health. 

## 2. Results

### 2.1. Identification of C. palmyrensis

A strain of *Coolia palmyrensis* (DISL57) was isolated from St. Thomas, U.S. Virgin Islands. The isolate was maintained in a laboratory culture (see methods) and confirmed by 28S rRNA gene sequence analysis as *C. palmyrensis* (GenBank Accession # OP392325). The LSU D1-D2 was used to determine the species of our strain, as is common for benthic dinoflagellate identification [37]. Our sequence had 100% coverage in existing entries for *C. palmyrensis* and 98.61% identity with prior database entries, e.g., GenBank Accession # MT295362.1. The next closest species match was with *C. santacroce*, also reported by [20] at only 84.43% identity to our sequence. The level of identity between our strain and existing *C. palmyrensis* sequences, compared to the significant interspecific genetic distances observed between *Coolia* species [20,38], provides support for our identification of this strain as *C. palmyrensis*. 

### 2.2. Bioactivity Associated with C. palmyrensis Media Extracts

Spent media from cultures of *C. palmyrensis* was extracted by adding HP-20 beads directly to an exponentially growing culture. Following incubation for 48 h, HP-20 was separated from cells and media and extracted in methanol (MeOH). The MeOH was dried, resuspended in 5% aq MeOH, and sequentially partitioned with ethyl acetate (EtOAc) and dichloromethane (DCM), yielding three partitioned fractions (EtOAc, DCM, and H_2_O). Each fraction was further separated by loading subsamples onto C18 solid phase extraction (SPE) cartridges and sequentially eluting with 10% aqueous (aq) MeOH, 60% aq MeOH, and 90% aq MeOH to yield three eluted fractions from each partitioned fraction. See Section 5.5. for methodological details on the extraction and separation process. A descriptive nomenclature is used throughout the rest of this manuscript to refer to different extract fractions. For example, H_2_O-C18-SPE-90% represents the fraction produced from loading the H_2_O fraction from the partition onto C18-SPE cartridges and capturing the 90% aq MeOH elute. 

**Figure 1:**
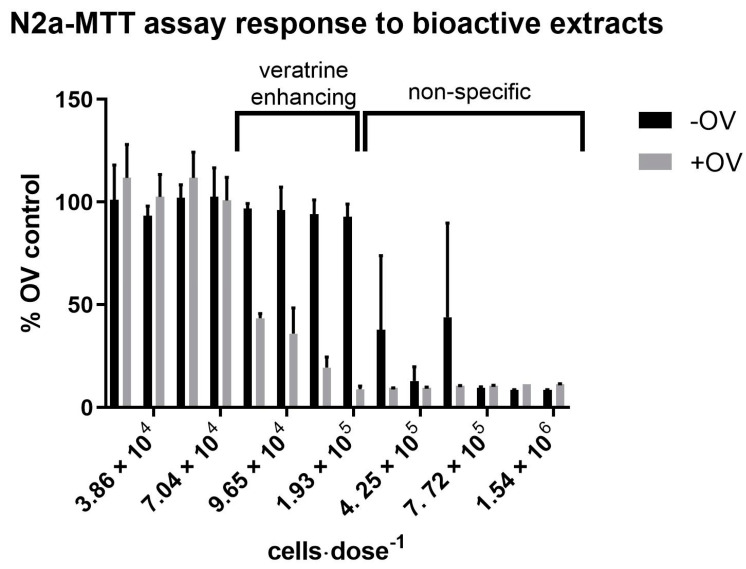
The effects of H_2_O-C18-SPE-90% fractions on the N2a-MTT assay. Lower doses (2.82 × 10^4^–7.04 × 10^4^ cells) exhibited no bioactivity. Mid-range doses (7.72 × 10^4^–1.93 × 10^5^ cells) exhibited veratrine enhancing (+OV only) bioactivity. Highest doses (4.22 × 10^5^–1.54 × 10^6^) exhibited non-specific (both +OV and −OV) bioactivity. Sample absorbance (% OV control) is a measurement that is inversely proportional to mitochondrial dehydrogenase activity, which is used as an indicator of cell function. See Figure S1 for a representative CTX3C standard curve.

Each extract fraction produced was tested for bioactivity using a N2a-MTT assay. Specifically, each sample extract was co-treated with and without a mixture of ouabain and veratrine (OV) present to evaluate “CTX-like” sodium channel specific (NaV) bioactivity and non-specific neurotoxicity, respectively. At doses between 3.86 × 10^4^ and 9.65 × 10^4^ cells·dose^−1^, the H_2_O-C18-SPE-90% fractions from the *Coolia* extracts exhibited a NaV-specific effect only, indicating the presence of compounds that enhance the toxicity of veratrine (Figure 1) is akin to CTXs [39]. At higher doses (i.e., 2.12 × 10^5^–7.72 × 10^5^ cells·dose^−1^), the same fractions exhibited non-specific bioactivity by affecting cells co-treated with and without OV (Figure 1). 

The H_2_O fractions (prior to SPE clean-up; Figure S2) from both *C. palmyrensis* media extracts and controls exhibited non-specific bioactivity on the N2a-MTT assay. After separation of the control samples via C18-SPE, the non-specific bioactivity was conserved in the 10% aq MeOH control, while the 60% and 90% aq MeOH controls exhibited no bioactivity/interference (Figure S3). 

Table 1 shows that none of the sample fractions from *C. palmyrensis* media or control DCM partitions exhibited bioactivity on the N2a-MTT assay. In contrast, the EtOAc partitions from both algae and control extracts exhibited non-specific cytotoxicity on the N2a-MTT assay, indicating matrix associated interference (Figure S4). This interference was further investigated by analyzing the residue from 300 mL of evaporated fresh HPLC grade EtOAc (i.e., the same volume used and pooled from partitioning). The fresh EtOAc exhibited similar non-specific cytotoxicity, indicating that the interference was derived from the solvent. When EtOAc residues were further separated by C18-SPE, the control associated interference occurred only in the 90% aq MeOH eluate (Figure S5). The 10% and 60% aq MeOH elutes from the algae extracts exhibited no response on the assay. It should also be noted that when EtOAc extracts and controls were added to the N2a-MTT assay, the phenol red pH indicator in the FBS-RPMI medium changed from red to yellow, which typically indicates that pH is lowered. 

### 2.3. Targeted LC-MS/MS Analysis of Coolia palmyrensis 

Fractionated *C. palmyrensis* extracts were analyzed via ultra-high-performance liquid chromatography (UHPLC) coupled to tandem mass spectrometry (MS/MS) in both positive and negative ion modes (based on optimized protocols) for multiple reaction monitoring (MRM) transitions of known algal toxins. Organic fractions of EtOAc and DCM were evaluated by targeted MRM of known lipophilic shellfish toxins, and H_2_O fractions were evaluated for the paralytic shellfish toxins (PST’s) and tetrodotoxins (TTX’s, see methods for full list of analytes). No MRM transition ion pairs for any of the targeted lipophilic shellfish toxins, PST, or TTX analogs were identified in any of the extract fraction from the algal media. 

### 2.4. Liquid Chromatography High Resolution Mass Spectrometry

**Figure 2:**
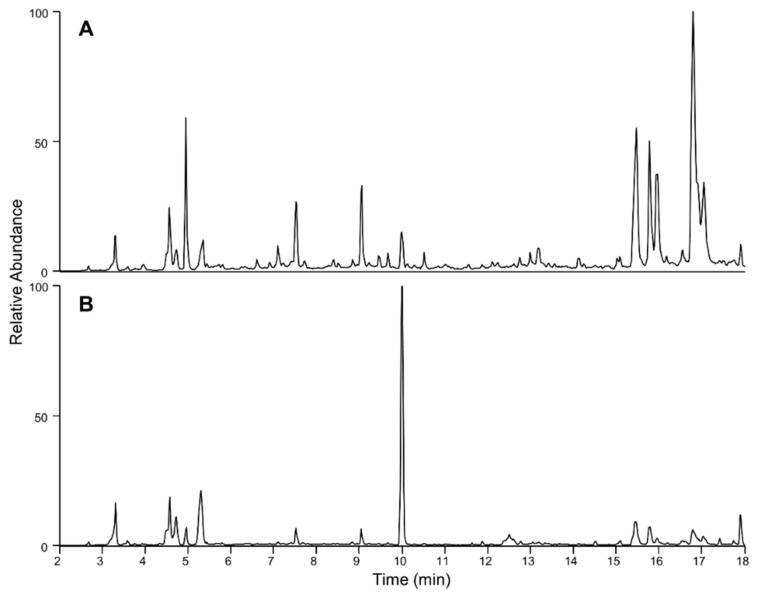
Total ion chromatograms (*m*/*z* 750–1400) of *Coolia palmyrensis* media H_2_O partition C18-SPE-90% fraction using LC-HR-MS with: (**A**) negative ionization and (**B**) positive ionization mode.

**Figure 3:**
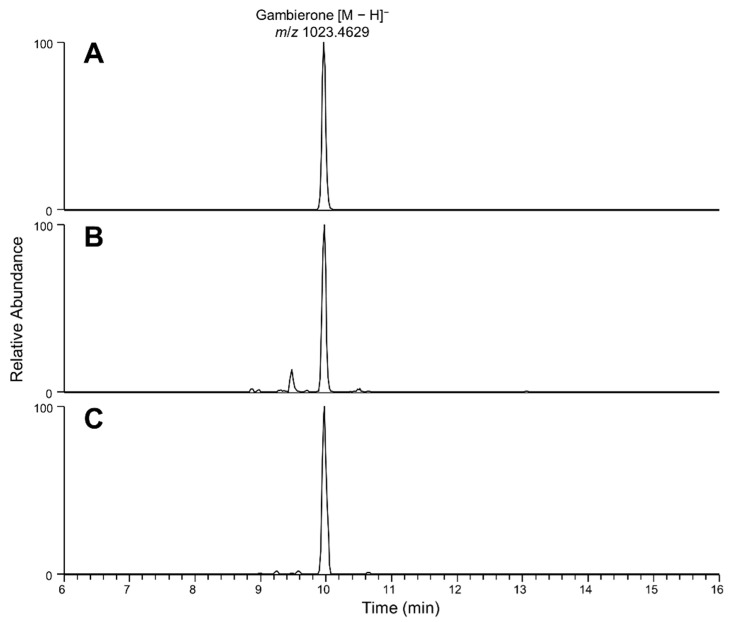
Extracted ion LC-HR-MS chromatogram of *m*/*z* 1023.4629 (±5 ppm) of (**A**) gambierone standard, (**B**) H_2_O fraction of *Coolia palmyrensis* media, and (**C**) H_2_O-C18-SPE-90% fraction in negative mode, which confirmed the presence of gambierone.

Full-scan LC-HR-MS chromatograms of the C18-SPE-90% media fraction remained complex with many peaks detected (Figure 2). Exact mass searches for known CTX and *Gambierdiscus-*related compounds were performed (with results shown in Figure 3), highlighting the presence of trace levels of gambierone ([M-H]^−^ *m*/*z* 1023.4656, 2.7 Δ ppm, 10 min) based on exact mass and retention time comparison with an authentic standard.

**Figure 4:**
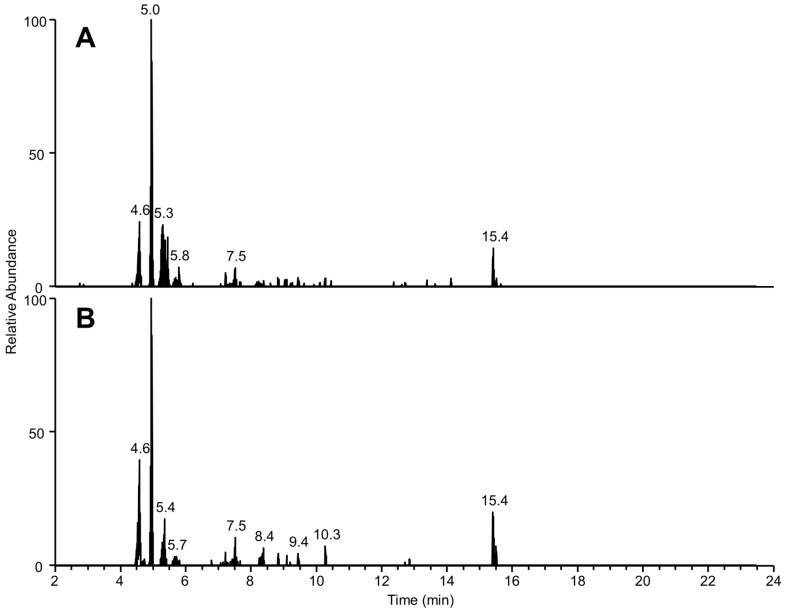
Extracted ion LC-HR-MS/MS chromatogram of (**A**) *m*/*z* 219.1378 and (**B**) *m*/*z* 233.1533 (±5 ppm) of *Coolia palmyrensis* media C18-SPE-90% fraction in positive mode DDA acquisition.

Given the complexity of this fraction, non-targeted LC-HR-MS methods were applied to evaluate the presence of possible compounds of interest. Data-dependent acquisition was used to acquire non-targeted fragmentation data for the dominant peaks in this fraction in both negative and positive ionization modes. Given the presence of gambierone and the CTX-like activity of this fraction, the data were screened for gambierone-like and CTX-like metabolites, such as ladder-frame polyethers. LC-HR-MS of gambierones highlighted dominant product ions at *m*/*z* 219.1378 or 233.1533 [40]. Extracted ion chromatograms were generated from the MS/MS spectra for these ions (Figure 4), revealing that six peaks produced these ions (Table 2). The molecular formulae of these peaks were estimated based on exact mass and isotope distribution. While the peak with an [M + H]^+^ at *m*/*z* 1275.8795 had a ring and double bond equivalent (RDBE) of two and is unlikely to have a polyether backbone, the others had a high RDBE, suggesting more complex backbone structures. Unlike gambierones, the product ions for these peaks (Table 2) were not dominant in the MS/MS spectra, and the lack of SO_3_ loss implies they are unlikely to be gambierone analogs. Another diagnostic feature in gambierone fragmentation is the neutral loss of SO_3_ (79.9563) in positive fragmentation, which was also extracted from the DDA acquisition. An additional peak with a precursor at *m*/*z* 1260.6594 was extracted from the chromatogram, which, upon further inspection, was a result of water losses from in-source fragmentation (Table 2); therefore, the molecular ion at [M-H]^−^ 1294.6643 (C_62_H_104_O_25_NS; 1.5 Δ ppm) provided a better estimate of the molecular formula. The presence of these peaks in the bioactive fraction suggests they may be linked to the bioactivity; however, further bioassay-guided fractionation and structural characterization are necessary. 

## 3. Discussion

### 3.1. Bioactivity and Gambierones in C. palmyrensis Media Extracts

The qualitative N2a-MTT assay used in this study is similar to that proposed by [39] and revised by others, e.g., [41]. The method incorporates the MTT assay to measure cellular mitochondrial dehydrogenase activity (MDA) as an endpoint and proxy for the viability of N2a cells pre-exposed to sample extracts. This method has been adapted by researchers studying CP to assess the toxicity of various fish and algae extracts contaminated with CTXs. Ciguatoxins act by specifically targeting site-five of voltage-gated sodium channels (NaVs), which enhances activation of the channel causing cellular depolarization at resting membrane potentials [42,43] but does not lead to neuronal death. These effects lead to persistent neurological changes in humans who have consumed seafood contaminated with CTXs. Alone, CTXs are not toxic to cultured N2a cells, but they enhance the persistent activation of NaVs and cytotoxicity caused by the steroidal alkaloid veratrine [44]. By pre-treating N2a cells with both veratrine (or the related compound veratridine, which has the same NaV mechanism) and the Na^+^/K^+^ ATPase inhibitor, ouabain (+OV), CTXs’ and brevetoxin’s (PbTX) activity can be assessed in a dose-dependent manner [39]. Running +OV and −OV treatments in parallel allows veratrine’s enhancing bioactivity to be distinguished from non-specific bioactivity that might be caused by other toxins or matrix interference [39,41].

In the *C. palmyrensis* media extracts, bioactivity was detected in the 90% aq MeOH SPE eluate from the partitioned H_2_O fraction. Doses ranging from 3.86 × 10^4^ to 9.65 × 10^4^ cells·dose^−1^ caused a reduction in MDA under +OV conditions only. This result indicates that the extracts enhance the toxicity of veratrine and are characteristic of extracts containing CTXs or PbTXs. Although the same fractions were not found to contain any previously described CTXs, PbTXs, or other NaV toxins via UHPLC-MS/MS and LC-HR-MS, they were found to contain gambierone. Gambierone has been shown to induce a hyperpolarizing shift in the voltage-dependent activation of NaV in human neuroblastoma cells (SH-SY5Y), indicating a similar effect on Na^+^ currents and hyperpolarized potentials as synthetically produced CTX3C [45]. Likewise, gambierone causes Na^+^ dependent increases in cytosolic Ca^2+^ in human cortical neurons, an effect also caused by CTX3C [46]. Despite these bioactivities, it has been demonstrated that gambierones alone will not kill SH-SY5Y cells [45] or human cortical neurons [46]. Additionally, in this study we found that standards of both gambierone and 44-methylgambierone had no effect on the N2a cell viability as measured in the N2a-MTT assay (Figure S6), aligning with prior studies [45,46,47]. From this, we hypothesize that both the NaV specific bioactivity and non-specific bioactivity observed in our samples can be attributed to a novel metabolite or suite of metabolites. 

In addition to finding gambierone in our extracts, LC-HR-MS analysis revealed that the semi-purified bioactive fractions were chemically complex and contained multiple peaks with exact masses suggestive of possible polycyclic ether cores based on mass defect and the loss of water and/or sulfate in the positive ion mode (Table 2). Veratrine-enhancing bioactivity was observed at doses between 3.86 × 10^4^ and 9.65 × 10^4^ cells·dose^−1^. However, higher doses of the same extracts (i.e., 2.12 × 10^5^–7.72 × 10^5^ cells·dose^−1^) exhibited non-specific bioactivity by reducing MDA in cells treated with and without OV. While matrix effects are well described at high cellular equivalent doses for the N2a-MTT and related assay variants, it is possible that some of the undescribed peaks detected via HR-MS represent novel toxins responsible for these observed bioactivities. Unfortunately, due to the limited sample, we were unable to further isolate and structurally elucidate these compounds. Future efforts will be focused on large scale isolation and purification of the sources of this bioactivity, which will also support efforts on their biological role. Other studies have reported that extracts from *Coolia* spp., including *C. palmyrensis,* exhibited non-specific cytotoxicity towards various cell lines. For instance, in a study by Karafas and colleagues [20], crude *C. palmyrensis* cell extracts were found to be toxic to Rhabdomyosarcoma cells and may reflect similar non-specific bioactivity to that in our *C. palmyrensis* media extracts. 

The Diaion^®^ HP-20 resin used in this study is a common tool for extracting algal toxins from media and seawater [36,48,49,50]. Despite its usefulness, HP-20 has been shown to exhibit interferences on various assays, including the N2a-MTT assay. For example, methanolic HP-20 extracts from control seawater have been reported to have non-specific effects on the N2a-MTT assay at concentrations of 121 ± 31 mg HP-20·mL^−1^ dry weight [36]. Hydrophilic fractions of partitioned HP-20 extracts from control seawater have also been shown to exhibit a non-specific effect at 1000 mg HP-20·mL^−1^ dry weight [49]. In this study, we used much greater quantities of HP-20 to ensure a thorough extraction of secondary metabolites from the media and generate a high yield of material. Process controls were also integrated into the workflow to distinguish potential HP-20 interference from novel bioactivity. Hydrophilic fractions from partitioned control extracts exhibited minimal veratrine-enhancing effects at 3 g HP-20 mL^−1^ and high veratrine-enhancing effects at 9.84 g HP-20 mL^−1^ (Figure S4). The NaV-specific veratrine enhancing bioactivity observed here is surprising, as other studies report only non-specific effects (i.e., +OV and −OV; [36,49]). The differences between our controls and those of other studies may be caused by our unique partitioning procedures and controlled assay conditions. Furthermore, the veratrine-enhancing effect may be caused by increased salts or Na^+^ concentrations in the control samples, which may exacerbate the NaV activating effects caused by veratrine. Following partitioning, our samples were loaded onto C18 SPE columns and eluted sequentially with 10%, 60%, and 90% aq MeOH. The corresponding control fractions were dosed at 34 g HP-20 mL^−1^ wet weight, which greatly exceeded that of our highest corresponding algal media extract doses of 6 g HP-20 mL^−1^ wet weight. Among the C18 separated control samples, only the 10% aq MeOH fraction exhibited bioactivity (Figure S2). It should be noted that the 10% aq MeOH fractions are expected to contain all the salts from the original hydrophilic fraction, whereas the 60% and 90% fractions are expected to contain minimal salt. Neither the 60% nor 90% aq MeOH fractions from control samples had an effect on the N2a assay, whereas the corresponding 90% aq MeOH fraction from our culture extracts exhibited veratrine-enhancing bioactivity. From this, we are confident that the bioactivity associated with the 90% aq MeOH was caused by algal metabolites and not the HP-20. Additionally, the C18 SPE clean up step appears to be useful in removing salts and other HP-20 associated interferences from samples. 

### 3.2. Role of Gambierones and C. palmyrensis in CP

The earliest descriptions of gambierone and the related 44-methylgambierone (first described as MTX-3) came from extracts of *G. toxicus* [51] and *Gambierdiscus belizeanus* [45], respectively. The production of these compounds by CTX-producing *Gambierdiscus* spp. in combination with their NaV specific bioactivity has raised concerns that gambierones contribute to CP symptoms. More recent studies have found gambierone analogs in extracts of most *Gambierdiscus* spp. and multiple *Fukuyoa* spp. and *Coolia* species [24,25,26]. Table 3 provides a summary of gambierone production in *Coolia*. Murray and colleagues [26] conducted a thorough evaluation of 44-methylgambierone production by *Gambierdiscus* and cohabiting dinoflagellates. Among the *Coolia* species tested, only *C. malayensis* and *C. tropicalis* were reported to produce 44-methylgambierone. In the same study, they reported that strains of *C. malayensis* from Australia (*n* = 3), New Zealand (two of seven), and Brazil (*n* = 1); [24] produced 44-methylgambierone, while strains from the Cook Islands (*n* = 3) or Hong Kong (*n* = 1) did not. Furthermore, none of the *C. canariensis*, *C. monotis*, or *C. palmyrensis* strains tested were found to produce 44-methylgambierone [26]. In a follow-up study, two strains of *C. malayensis* were reported to produce both gambierone and 44-methylgambierone, while two strains of *C. tropicalis* produced 44-methylgambierone but only contained trace amounts of gambierone [25]. 

Ciguatoxins, the causative agents of CP, act by activating NaVs causing cellular depolarization, spontaneous nerve firing, and alteration of the resting membrane potential of the cell. This results in a plethora of neurological symptoms that can manifest in people who have consumed seafood contaminated with CTXs [44]. To date, only a handful of studies have assessed the toxicity of gambierones. However, the biological systems used in these assessments have been only loosely relevant to CP. For example, when injected intraperitoneally in mice, gambierone exhibited an LD50 of 2.4 mg/kg [25] and 44-methylgambierone was estimated to be one tenth as potent as gambierone [26]. Additionally, gambierone has been reported to alter Na^+^ currents and Na^+^-dependent Ca^2+^ currents in SH-SY5Y cells, although to a lesser extent than CTX3C [25,45]. For example, gambierone induces a hyperpolarizing shift in the voltage-dependent activation of NaV in human neuroblastoma cells (SH-SY5Y), indicating a similar effect on Na^+^ currents and hyperpolarized potentials as synthetically produced CTX3C [45]. Likewise, gambierone caused Na^+^ dependent increases in the cytosolic Ca^2+^ of human cortical neurons, an effect also caused by CTX3C [46]. Despite these bioactivities, it has been demonstrated that gambierones alone will not kill SH-SY5Y cells [45] or human cortical neurons [46]. Additionally, direct assessment of these compounds during our investigations showed that gambierone and 44-methylgambierone had no effect on mouse neuroblastoma cells via the N2a-MTT assay (Figure S6; consistent with others [47]), suggesting that their mode of action is quite different to the CTXs. Although gambierones did not have an apparent effect on the sodium channel-specific N2a-MTT assay, they cannot be ruled out as contributors to the neurological symptoms associated with CP in humans until their toxicokinetics and molecular targets are fully characterized. For example, any contribution of gambierones to CP symptoms would likely arise from oral consumption. Therefore, studies assessing toxicity via alternative routes of exposure or in vitro cell and tissue assays must employ caution when extrapolating the potency of gambierones to CP. Furthermore, CTXs may undergo structural modifications that can increase or decrease their toxicity [51], and this would need to be further evaluated for the gambierones. Also, the sensitivity/bioactivity of CTXs varies between NaV isoforms, which may be differentially expressed between different tissues and organisms [52]; this likely applies to gambierone as well. Data regarding the bioaccumulation, biotransformation, and toxicology of gambierones is needed to fully understand whether these compounds contribute to CP or any toxicological effects both in vitro and in vivo. 

### 3.3. Extracellular Release of Metabolites into C. palymyrensis Media and Potential Ecological Implications

Multiple studies have reported that *Coolia* cell extracts were toxic to the larvae of various organisms, including *Artemia* spp. [27,28,29,30]; the sea snail *Haliotis virginea* [27]; sea urchin *Heliocidaris crassispina* [28]; and marine medaka *Oryzias melastigma* [31]. However, the exposure of larvae to dissolved cell extracts does not consider all relevant exposure pathways. For example, organisms are most likely exposed to cellular metabolite pools via consumption or grazing, where dissolved metabolites from cell extracts would likely enter exposed organisms through the gills or external membranes. In this study, we found that the extracts of *C. palmyrensis* DISL57 culture medium contained the polyether gambierone and a suite of other polyether-like metabolites that collectively caused both veratrine-enhancing activity (unrelated to gambierone) and non-specific cytotoxicity that could be related to novel metabolites. This indicates that non-dietary routes of exposure may play an important role in the toxicity of *Coolia* to cohabiting microfauna. The extracellular release of metabolites and bioactivity have been reported in other dinoflagellate species. For example, *Gambierdiscus pacificus* (strain G10DC) exhibited similar magnitudes of CTX-like bioactivity in cell and media extracts [36]. Strains of the planktonic dinoflagellates *Alexandrium catanella* and *A. tamarense* have also been reported to release metabolites into the growth medium, which induced cell lysis in various planktonic flagellates [35]. Additionally, solid phase adsorption toxin tracking (SPATT) devices have been used to assess a plethora of dissolved algal toxins in situ, including the lipophilic pectenotoxins and okadaic acids [48,50], CTXs [36,49], and the hydrophilic domoic acid and saxitoxins [50].

The extracellular release of bioactive metabolites of *C. palmyrensis* could have important ecological implications. However, it is important to consider how the metabolites may have ended up in the media. For example, many dissolved toxins collected in situ on SPATT disks are thought to do so following cell lysis [50]. In this study, less than 1% of cells were lysed or exhibited ecdysis indicating that the observed bioactivity and metabolites were released from healthy dinoflagellate cells. Some genera excrete toxins from the cell into mucus, e.g., the filtrate from a strain of *A. pseudogonyaulax* was reported to have no lytic effects [35], while in co-culture, cells were lysed after being trapped in mucus produced by *A. pseudogonyaulax* [35]. Similarly, direct contact with *Ostreopsis ovata* cells and mucilage had toxic effects towards the marine invertebrate larvae *Artemia salina*, *Tigriopus fulvus*, and *Amphibalanus amphitrite* [34]. A follow-up study by the same research group showed that exposure of *A. salina* to mucus from *Ostreopsis* c.f. *ovata* resulted in toxic effects [33]. Contrarily, 0.22 µm media filtrates representing dissolved toxins had little to no toxic effect on *A. salina*, indicating that the mucus was an important conduit to the toxicity observed in the marine invertebrates tested [33]. The investigators hypothesized that toxigenic mucus likely reduces grazing pressures and potentially facilitates mixotrophy by the producing dinoflagellates. In this study, we identified veratrine-enhancing and non-specific neurotoxicity from the dissolved metabolite pool in the media of *C. palymyrensis* following gentle removal of the dinoflagellate mucus. While we are confident that the dissolved metabolite pools were released from live and healthy cells, it remains unclear as to whether these metabolites were actively secreted from the cell or exited the cell by diffusion. The dissolved nature of this bioactivity indicates that these compounds have the potential to reach extracellular targets via diffusion or enter the marine food web through non-dietary routes of exposure, i.e., dermal or via gills. Once metabolites are elucidated, future studies will focus on the effects of these metabolites on microinvertebrate grazers and other members of the food web and whether they facilitate reduced grazing pressures or mixotrophy by *Coolia*. Tropical epibenthic communities harbor a variety of toxin producing genera, so co-existing microfauna are likely exposed to complex profiles of dissolved dinoflagellate metabolites, the effects of which may be synergistically enhanced. This could have important ecological implications regarding the fitness and recruitment of natural cohabiting epibenthic microfauna, which could influence top-down grazing pressures on *Coolia* and other cohabiting dinoflagellate populations.

The effects that dissolved metabolites have on exposed organisms largely depends on their in situ concentrations [49]. Environments harboring *Coolia* and other benthic dinoflagellates are often subject to advective forces from currents and waves, which can distribute and dilute dissolved metabolites. However, these processes are muffled by the benthic boundary layer, the three-dimensional structure of macroalgal thalli, and dense vegetative canopies. The reduced flow around and within the thalli of host macroalgae (i.e., especially during periods with reduced currents and wave influence), reduces the advective export of dissolved constituents [53], including extracellular dinoflagellate metabolites. The result is a thallus-bound habitat (i.e., thallusphere) that is physically semi-isolated and chemically distinct [54] from the water column during periods of low turbulence (e.g., slack tide and/or periods of minimal wave influence). It is anticipated that during these periods, dissolved pools of bioactive metabolites from dinoflagellates, such as *Coolia*, may reach ecologically relevant concentrations. 

In addition to ecological function, the exposure of marine organisms to extracellular pools of dinoflagellate toxins may lead to bioaccumulation through alternative exposure pathways (i.e., gills) and play an important role in organismal or human health-related issues, such as CP. To date, most studies attempting to assess the flux of benthic dinoflagellate toxins into the food web only consider intracellular toxin pools. However, mounting evidence suggests that extracellular pools represent a significant proportion of the total toxins produced [36,55]. In addition to dissolved toxins in seawater (or culture medium), the large polycyclic ether backbone of many algal toxins possesses hydrophobic regions that cause the compounds to localize and adsorb on hydrophobic surfaces [44]. This has been demonstrated for multiple PbTX congeners, which were found to localize to the surfaces of various microalgae, microfauna, and even macrophytes [56]. Benthic epiphyte communities may represent another important pool of extracellular dinoflagellate toxins that may contribute to the flux of toxins into the food web. 

## 4. Conclusions

In this study, we investigated the extracellular occurrence of metabolites and bioactivity of *Coolia palmyrensis* DISL57. To do so, the culture media was extracted with HP-20 resin and semi-purified fractions of those extracts were assessed using a combination of bioactivity, i.e., N2a-MTT assay, targeted chemical analysis UHPLC-MS/MS, and non-targeted LC-HR-MS. Both veratrine-enhancing and non-specific bioactivity was identified in the water-soluble fractions of media extracts. Gambierone was identified in the same extracts via LC-HR-MS analysis, but based on the lack of activity by gambierone and 44-methylgambierone on the N2a-MTT assay, these compounds were not the likely cause of the novel bioactivity observed in our samples. In addition to gambierone, the same fractions were found to contain multiple peaks with exact masses suggestive of potential polycyclic ether structures that may represent novel bioactive algal metabolites. However, more work is needed to identify the source of the observed bioactivity following large scale culture and purification. 

These findings highlight *Coolia* as a source of dissolved and bioactive metabolite pools that other cohabiting epiphytes are anticipated to be exposed to in situ. Furthermore, this is the first report of the NaV-specific bioactivity being associated with *Coolia* extracts, indicating that these compounds may apply physiological stressors to exposed microfauna. Future work is needed to assess the ecological relevance of these extracellular metabolite pools and their associated bioactivity. 

## 5. Materials and Methods 

### 5.1. Materials, Reagents, and Glassware

All glassware used for culturing and sample preparation was precleaned by washing with laboratory soap and soaking for 1 h in 10% HCl. Glassware was then rinsed three times, alternating between laboratory grade MeOH and reverse osmosis H_2_O. 

Algal culture media was a modified version (i.e., without Tris or Si) of the recipe from [57]. Artificial seawater (ASW) was prepared with Instant Ocean^®^ Sea Salt to 35 ppt. Nutrients were purchased in a Kellers (K) media kit (Bigelow National Center for Marine Algae and Microbiota, East Boothbay, ME, USA) and added to ASW to the specified proportions. Media was filter sterilized under vacuum through a 45 mm diameter, 0.2 µm pore size cellulose acetate filter.

Ouabain octahydrate (O) and veratrine hydrochloride (V) were from Sigma Aldrich (St. Louis, MO, USA). The 3-[4,5-dimethylthiazol-2-yl]-2,5-diphenyl-tetrazolium (MTT) was purchased from Alfa Aesar (Haverhill, MA, USA) and prepared in sterile phosphate buffered saline (PBS) from Medicago (Quebec City, QC, Canada). HPLC grade dimethyl sulfoxide was purchased from Fisher Scientific (Waltham, MA, USA). Ciguatoxin3C (CTX3C) was purchased from Wako Chemicals (Osaka, Japan) and a 50 ng mL^−1^ stock was prepared in LC-MS grade MeOH, aliquoted and sealed in amber vials, and stored at −20 °C until use. Gambierone (19.9 μg mL^−1^ in MeOH) and 44-methylgambierone (19.9 μg mL^−1^ in MeOH) was purchased from Cifga (Lugo, Spain).

Murine neuroblastoma cells (Neuro-2a; ATCC CCL-131) were purchased from the American Tissue Culture Collection (Manassas, VA, USA). Mycoplasma-free Neuro-2a cells were modified (i.e., OV desensitized) and lines were generated and maintained to ensure maximal and stable cell response to CTX in MTT-based assays prior to use (available on request; see [57]). Powdered Roswell Park Memorial Institute (RPMI) 1640 medium (Millipore Sigma, Burlington, MA, USA) was prepared in 10 L batches with sterile ultrapure water (18 mΩ) and 1L aliquots filtered (polyethersulfone 0.2 µM Supor membrane; Pall Corp; Port Washington, NY, USA) into sterile bottles. Supplements included sterile L-glutamine (200 mM stock), sodium pyruvate (100 mM stock), and heat-inactivated fetal bovine serum (all from Gibco; Grand Island, NY, USA). Trypsin-EDTA (0.025% stock) used in cell detachment and harvest was from Corning (Corning, NY, USA). Cell culture consumables, including serological pipettes, tubes, flasks, and micro-well plates, were from CellTreat^®^ (Shirley, MA, USA). Trypan blue (Fisher Scientific, Waltham, MA, USA) was prepared to 0.2% in sterile PBS (pH 7.4) and used in cell enumeration and viability assessments. 

Diaion™ HP-20 resin was purchased from Fisher Scientific (Waltham, MA, USA). All solvents used in sample preparation were HPLC grade and purchased from Fisher Scientific (Waltham, MA, USA) and include MeOH, dichloromethane (DCM) and ethyl acetate (EtOAc). Bond elute C18 SPE cartridges (3 mL, 1 g) were from Agilent Technologies (Santa Clara, CA, USA). All solvents and reagents used for MS analysis were LC-MS grade and include MeCN, MeOH, ammonium fluoride, formic acid, and ammonium hydroxide, purchased from Fisher Scientific (Waltham, MA, USA). 

### 5.2. Single Cell Isolation and Culture Establishment

Single dinoflagellate cells identified as *Coolia* spp. were isolated from live field samples collected from Black Point on the south side of St. Thomas, U.S. Virgin Islands, in December 2017. Macroalgae substrate samples (~200 g) consisting of a combination of the most abundant macroalgae genera were collected on Scuba at a depth of approximately 20 m. Samples were gently agitated in 1 Gal Ziploc bags with approximately 2 L of 10 µm filtered ambient seawater to pool epiphytes. To separate dinoflagellate cells from larger particles and microfauna, the seawater-containing epiphytes were filtered sequentially through sieves with 200 µm and 20 µm nitex mesh. This process of shaking and sieving was repeated five times and the contents captured on the 20 µm sieve was subsequently back-rinsed into a polypropylene tube and made up to a volume of 50 mL with filtered seawater. A subsample (1 mL) of the well mixed suspension was added to 19 mL modified K/50-media in 25 cm^2^ tissue culture flasks with filter-caps providing the final live sample. Live samples were shipped overnight to the Dauphin Island Sea Lab in Alabama, USA. Single cells were isolated from live samples with capillary pipettes, placed onto a sterile microscope slide, and rinsed five times in fresh media. Following the final rinse or until the cell was visually isolated from other particles, cells were deposited into a single well of a 24-well culture plate containing 2 mL modified K/2 medium. Following 1–2 weeks of growth, isolated cells were transferred to 15 mL culture tubes.

### 5.3. Dinoflagellate Culture Conditions

During general maintenance, monoclonal cultures were grown at 25 °C on a 12:12 h light:dark cycle in 15 mL borosilicate screw cap culture tubes containing 6 mL of media. Irradiances during the light cycle were maintained at ~70 µmol photons·m^−2^·s^−1^. The culture medium used was a modified version of the recipe from [58], in which Tris and silica were excluded. Net photosynthesis in culture results in a raised culture pH over time, so Tris buffer was excluded from the recipe. Silica is not required by dinoflagellates, and the absence of silica in culture can help prevent contamination by faster growing diatom species. Constituents of the Keller recipe were added to artificial seawater (ASW) prepared to a salinity of 35 ppt with Instant Ocean^®^ salts and then filter sterilized through a 0.22 µm cellulose acetate filter.

To scale up culture volume, 6 mL cultures were transferred to 15 mL of fresh medium in 50 mL borosilicate culture tubes. Once the cultures’ densities in 15 mL volumes reached >2000 cells·mL^−1^, the entire volume was transferred to sterilized 500 mL medium in 500 mL borosilicate culture bottles. Upon inoculation to 500 mL volumes, cultures were placed under down-facing, cool, white fluorescent lamps, where constant photon flux densities at the surface of the culture were approximately 75 µmol photons·m^−2^·s^−1^. After 15 days of incubation, 500 mL cultures were expanded to 2 L volumes in 2.8 L Fernbach flasks and maintained under the same conditions. Prior to and in between extraction with HP-20, sub-samples were observed under the microscope to confirm typical cell morphology, flagellar movement, and behavior as proxies for culture health. Additional sub-samples (3 × 1 mL) were collected and preserved by adding 10 µL of 50% glutaraldehyde and stored at 4 °C for cell counts. Complete growth curves were not measured as a time course during this batch culture due to the experimental setup (described in Section 5.4.). However, since cultures were incubated for up to a maximum of 15 days in a 2 L volume following inoculation, with prior growth rates estimated at 0.25–0.30 and >99% of cells being viable at the time of harvest based on microscopic evaluation, we felt confident that the culture was in exponential to late exponential growth at the time of harvest. 

### 5.4. Dinoflagellate Species Identification

DNA was extracted from a cell pellet derived from 15 mL of culture. In 1.5 mL centrifuge tubes, cells were washed twice by resuspending in 2 mL PBS. Following rinsing, cells were pelletized by centrifugation at 300× *g*. Cell pellets were resuspended in 500 µL CTAB extraction buffer (2% CTAB, 0.1 M Tris base, 0.02 M EDTA, 1.4 M NaCl, 1% PVP-40) adjusted to a final pH of 5. Cells in CTAB were thoroughly vortexed and incubated for 15 min in a hot plate pre-heated to 60 °C. After incubation, cells debris was spun down at 14,000× *g*, and the supernatant was carefully removed and transferred to new 1.5 mL centrifuge tubes. A total of 5 µL of RNase solution A was added to the extract and incubated for 20 min at 37 °C. Following incubation, 500 µL of chloroform/isoamyl alcohol (24:1) was added to the extracts, vortexed for 5 s, and centrifuged for 1 min at 14,000× *g* to separate the phases. The upper aqueous phase was transferred to clean 2 mL centrifuge tubes, and DNA was precipitated by adding 420 µL of isopropanol and was allowed to chill overnight at −20 °C. Precipitated DNA was pelleted by centrifuging at 14,000× *g* for 10 min. The isopropanol was carefully removed, and the pellet was rinsed with 500 µL of ice-cold 70% ethanol to remove remaining salts from the samples. Ethanol was removed, and pellets were allowed to dry at room temperature overnight. Dried DNA pellets were redissolved in 50 µL sterile MilliQ H_2_O. A Thermo Scientific™ Nanodrop™ ND-1000 UV/vis spectrophotometer was used to measure absorbance of extracts at 260 nm as a proxy for DNA concentration and the ratios of absorbance at 260/280 and 230/260 as indicators of DNA purity.

The D1-D3 fragment of the LSU rRNA was amplified using primers D1R-F [59] and D3B-R [60]. The PCR amplifications were carried out in 50 µL reaction volumes containing 5 µL 10× Taq buffer, 1 µL of 10 mM dNTP mix, 5 µL of 25 mM MgCl_2_, 0.25 µL Taq DNA Polymerase (1.25 U), 32.75 µL nuclease free water, 0.5 µL forward primer, 0.5 µL reverse primer, and 5 µL template DNA. Thermocycling conditions for the D1-D3 fragment consisted of an initial denaturing step of 95 °C for 4 min, 35 cycles of 95 °C for 30 s, 60 °C for 2 min, and a final extension step of 72 °C for 10 min. Forward and reverse amplicons were subsequently Sanger sequenced (GENEWIZ^®^). Forward and reverse sequences were aligned using Geneious Prime^®^ and conflicts were resolved via manual inspection. Trimmed sequences were used as a query in BLAST (i.e., blastn) of the GenBank non-redundant (nr) database.

### 5.5. Isolation, Extraction, and Fractionation of C. palmyrensis from Large Scale Culture

Dissolved metabolites were extracted from the media of living *C. palmyrensis* cultures using Diaion ^®^ HP-20. Prior to use, HP-20 (20 g) was activated by saturating the porous surface of the resin with MeOH through gentle continuous mixing for 24 h in 200 mL HPLC grade MeOH (10 mL/g resin). This process saturates the surface of the resin with an aqueous solution to increase the interactions between dissolved compounds and the surface of the resin. Activated HP-20 was removed from the MeOH by filtering under light vacuum onto a 125 mm diameter 20–25 µm pore size Whatman™ #4 filter paper in a Büchner funnel. To remove any excess MeOH, the HP-20 still in the Büchner funnel was rinsed by resuspending in 200 mL sterile Milli-Q water which was then removed by applying light vacuum. This process was repeated twice for each batch of 20 g of HP-20. Once water is removed following the second rinse, loose HP-20 (i.e., ~20 g) was added to 2 L exponentially growing algal cultures. Cultures containing activated HP-20 were incubated for 24 h prior to harvesting. Following 24 h of soak time, HP-20 was removed from cultures by carefully pouring the cultures through a 10 cm diameter glass funnel containing a 100 µm pore size nylon mesh filter. This pore size allowed the media and most of the algal cells (~60 µm diameter) to pass through into a new culture vessel, while the HP-20 (>250 µm diameter) was captured. This method was tested prior to use to ensure that cells were not lysed during this process or held up in the HP-20. The harvested HP-20 was thoroughly rinsed with Milli-Q water to remove excess salts, spooned into a glass bottle, and stored in MeOH at −20 °C for later extraction. Following removal of HP-20, cultures were placed into the incubator for an additional 48 h and then the media was reextracted with HP-20. Each culture was extracted in this manner a total of three times, with the first extraction beginning 6 days after transfer to 2 L volume. The three extractions took place over 9 days following the first addition of HP-20, resulting in a total culture period of 15 days. The HP-20 from each extraction was pooled totaling 60 g. HP-20 (60 g) from duplicate 2 L cultures were subsequently pooled to make a single sample representing 4 L of media (120 g HP-20 per sample). Harvested HP-20 was rinsed into glass bottles and stored with MeOH at −20 °C until later extraction. Following extraction, aliquots of media containing live cells were examined for typical morphology and movement. 

Sequential extraction of the same culture and cells with fresh HP-20 provided a cumulatively greater metabolite pool than if we discarded the cultured cells afterwards. However, this experimental choice then limited our ability to quantify the toxin yield per cell in parallel since the addition of HP-20 is expected to modify the kinetics of metabolite release on a concentration gradient from presumably high concentration in the cell to the low concentration in the media (maintained low with repeated HP-20 capture). This process thus remained qualitative, akin to many large-scale isolation strategies in the hope of identifying target metabolites from bulk capture, that could be later tracked through all phases (i.e., cells, media, HP-20). The successive addition of HP-20 to the cultures through time also made estimations of biotic and abiotic degradation difficult, making subsequent estimates of the production or release of bioactivity or metabolites by the cells in culture only qualitative. However, cell counts were conducted for two of the eight replicate 2 L cultures to approximate the per cell contribution to bioactivity and are provided for qualitative assessment (See Section 5.6). We will seek to resolve these experimental limitations in future studies.

To extract metabolites from harvested HP-20, the resin was suspended in 250 mL HPLC grade MeOH and gently agitated for 2 h. The MeOH extract was collected by filtering onto Whatman (#4) as described above. The filtered HP-20 was rinsed twice with an additional 100 mL MeOH while still in the Büchner funnel to remove any residual extract. Methanolic extracts (e.g., both from cells and HP-20) were stored at −20 °C for at least 8 h to precipitate proteins. Chilled extracts were then filtered through a 125 mm diameter 2.5 µm pore size Whatman™ #5 filter paper to remove precipitates. Filtered MeOH was quantitatively transferred to a 500 mL borosilicate round bottom flask and evaporated via rotary evaporation at 40 °C. Dried extracts were resuspended first by adding 5 mL of MeOH and manually swirling the MeOH over the entire inner surface of the flask. Milli-Q^®^ H_2_O (45 mL) was added to each extract, then sonicated for 2 min until all visible residue was liberated from the surface of the flask. The resuspended extracts were quantitatively transferred to a 500 mL separatory funnel by rinsing the round bottom flask twice with 25 mL of ultrapure H_2_O (i.e., final volume = 100 mL of 5% aq MeOH).

**Figure 5:**
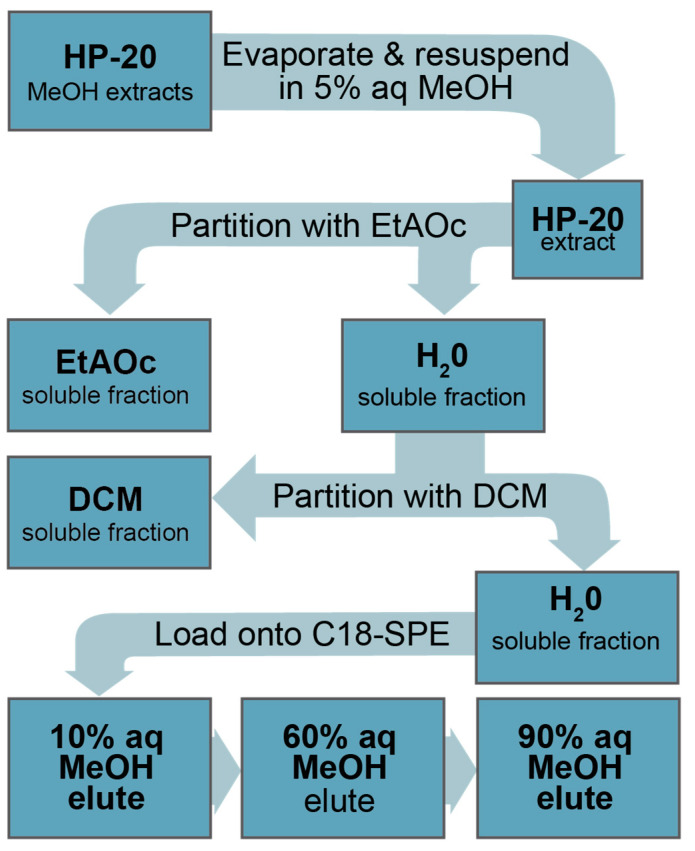
Liquid–liquid partitioning sequence for HP-20 extracts of *Coolia palmyrensis*.

Aqueous extracts were partitioned sequentially with EtOAc and then DCM, yielding three fractions (Figure 5). Partitioning with each solvent was performed three times in a 1:1 water:solvent ratio and combined, totaling 300 mL of solvent for each fraction. Following partitioning, solvents were removed via rotary evaporation (i.e., organic at 40 °C and aqueous at 45 °C), redissolved, and quantitatively transferred to 10 mL glass tubes. Final samples were evaporated to dryness at 40 °C under a steady stream of high-purity N_2_ gas. 

Once secondary extracts (e.g., from the liquid–liquid partition) were analyzed for N2a-MTT bioactivity, the remaining extract was further separated into three tertiary extracts (i.e., 10%, 60%, and 90% aq MeOH eluates) via a solid-phase extraction (SPE) flash chromatography sequence (Figure 5). Flash chromatography was performed using 1 g Agilent Bond Elute reverse-phase C18 SPE columns. SPE columns were pre-conditioned by first passing through 6 mL MeOH and then 6 mL of 10% aq MeOH. Secondary extracts produced from liquid–liquid partitioning were resuspended in 1 mL MeOH and sonicated for 5 min to facilitate dissolution. The 1 mL extract was then diluted to 10 mL with 18.2 MΩ-cm Milli-Q water to achieve a 10% aq MeOH solution. Following conditioning, samples in 10 mL of 10% aq MeOH were loaded onto the pre-conditioned C18 column. Loaded samples were then eluted sequentially, first with 5 mL 10% aq MeOH, which was combined with the loading elute, 5 mL 60% aq MeOH, and 5 mL 90% MeOH. The resulting tertiary fractions were then dried under a steady gentle stream of N_2_ gas while sitting in a water bath maintained at 45 °C until dry. 

### 5.6. Algal Cell Counts and Dosing

Six cultures (named cultures A–F) were used during process development (i.e., semi-purification and bioactivity isolation) for this study. Samples for cell counts were not collected from these cultures. Rather, doses relating to volume of *C. palmyrensis* culture were assessed. Once the bioactivity of extracts was confirmed, the experiment was repeated on two additional cultures (i.e., G and H). To provide rough toxicity·cell^−1^ estimates, triplicate sub-samples (1 mL) were collected from cultures G and H and counted using a Sedgewick-Rafter counting chamber. Triplicate counts were averaged and used to calculate total cells·culture^−1^ values and cells·dose^−1^ (i.e., total cells·culture^−1^ × the % of extract to assay well). Following a 15-day culture period and extraction with HP-20, cultures G and H were confirmed to contain less than 1% ruptured cells or cells exhibiting ecdysis. All partitions were regarded as 100% of the total extract. If 50% of a partition was further separated by SPE, each of the three resulting tertiary extracts (e.g., combined 10%, 60%, and 90% aq MeOH eluates) were said to represent 50% of the total extract. 

### 5.7. N2A-MTT (N2a) Assay

The assay in this study used a mouse neuroblastoma neuro-2a (N2a) cell line purchased from ATCC that was later modified for increased resistance to veratrine toxicity, allowing an improved dose–response range for bioactivity assessment as previously described [57]. During general maintenance, N2a cells were cultured in 75 cm^2^ tissue culture flasks containing 15 mL of RPMI-1640 media supplemented with 5% (*v*/*v*) heat-inactivated fetal bovine serum (FBS), 1 mM sodium pyruvate, and 1mM-glutamine. Culture flasks were maintained in a water jacketed humidified incubator at 37 °C and 5% CO_2_. Cell passaging was conducted routinely every 48 h, and flasks were re-inoculated with ~1,600,000 cells to maintain a steady growth rate. 

For the assay, N2a cells harvested during passage were seeded into 96-well plates with each well receiving 200 µL of fresh FBS-RPMI media containing ~30,000 cells. Seeded plates were incubated for ~18 h prior to dosing to allow the cells to form synaptic connections and adhere to wells. To differentiate between Na^+^ channel specific and non-specific bioactivity, each sample was dosed into six wells total. Three wells were dedicated to the assessment of CTX-like sodium channel activity (sensitized), and three wells for non-specific neurotoxicity (non-sensitized). To sensitize cells, 20 µL of PBS containing 2.5 mM ouabain and 0.25 mM veratrine were added (i.e., with OV). The veratrine acts by binding to and activating the NaV, which is otherwise inactive in N2a cells. Ouabain inhibits the function of the sodium–potassium ion pump, further exacerbating the effects of compounds that modulate the Na^+^ channel. In the presence of compounds exhibiting the same mode of action as CTX or PbTX, OV-treated cells will experience an increased flux of Na^+^ ions into the cell. The increased flux causes cellular depolarization resulting in mortality. The triplicate wells that do not receive the OV treatments (i.e., −O/V) represent any bioactivity (cytotoxicity) not specific to NaV activity and receive 20 µL of PBS to control for volume. Controls for +OV and −OV treatments consisted of eight wells each and were treated in the same manner as the sample wells. Once the OV and PBS are added to both the sample and control wells, sample wells each received 10 µL of sample redissolved in 5% FBS-RPMI (total well volume 230 µL). Dosed cells were incubated at 37 °C and 5% CO_2_ for ~18 h prior to development. 

Following the incubation, culture medium was removed from wells and 60 µL of MTT FBS-RPMI solution (i.e., 5 mg·mL^−1^ MTT in PBS with 5% FBS-RPMI in a 1:5, MTT:5% FBS-RPMI ratio) was added. Plates were then incubated for an additional 30 min. The MTT FBS-RPMI solution was subsequently removed from the wells, plates blotted dry, and 100 µL of dimethylsulfoxide (DMSO) added to solubilize the formazan precipitate formed in the presence of active mitochondria. Plates were shaken for 5 min and the absorbance immediately read at 570 nm (Sunrise^TM^, TECAN, Morrisville, NC, USA). 

The presence of OV in +OV assay wells was designed to slightly reduce the cell viability relative to −OV wells (approx. 15–25%) so that activity and response could be verified. To account for this, raw absorbance of sample wells was converted to % of the corresponding −OV or +OV control wells. 

To conserve extract for LC-HR-MS and UHPLC-MS/MS analysis, a full dose–response curve was not attempted in a single assay. Rather, the relative toxicity was assessed by assaying algal extracts alongside a duplicate full CTX3C dose–response curve. The pg CTX3C·dose^−1^ (i.e., x-values) was log transformed and a logistic non-linear four parameter variable slope model was fit to the data using Graphpad Prism software v7-9 (Boston, MA, USA). The models were used to extrapolate dose in pg CTX3C·dose^−1^ from algal extract doses with % absorbance values between the 80% and 20% linear portion of the corresponding CTX3C curves. To identify doses falling within this range while attempting to conserve material, doses were determined through a series of five assays, where a single target dose was then determined from the results of the previous assay. Cell toxicity estimates were calculated by dividing the pg CTX3C·dose^−1^ values from extracts to their corresponding cell·dose^−1^ values. 

Since gambierone was identified in the media extracts, the effect of gambierone and 44-methylgambierone was tested on the N2a–MTT assay to determine if the bioactivity observed could be attributed to these compounds. Aliquots of standards were dried and diluted in 200 µL 5%FBS-RPMI media, then serially diluted in the following ranges: gambierone 2.2 µg mL^−1^ to 17 pg mL^−1^; 44-methylgambierone 2.3 µg mL^−1^ to 17 pg mL^−1^, and dosed to the standard N2a–MTT assay format with and without OV as previously described (including all previously stated controls). Each complete dilution series was evaluated twice in triplicate over two cell lines, representing a total of four independent assays. The log dose was plotted in ng mL^−1^ and compared to duplicate CTX3C curves performed in parallel.

### 5.8. UHPLC-MS/MS Targeted Phycotoxin Analysis

Targeted analyses for lipophilic toxins (method A) were performed on Waters Xevo TQ and TQ-S triple quadrupole mass spectrometers equipped with electrospray ionization (ESI) probes and operating in MRM modes, coupled with Acquity UPLC systems (Waters, Manchester, UK). Chromatographic separation utilized a Waters BEH C18 1.7 µm column (50 × 2.1 mm) equipped with matching VanGuard and pre-filter and maintained at 30 °C. For lipophilic toxin analysis, mobile phase (MP) A was water and MP B was 90% aqueous acetonitrile, both containing 0.1% ammonium hydroxide solution, with a gradient from 15 to 100% B performed over three min, and an injection volume of 5 µL. Analytes targeted in these analyses included okadaic acid (OA), dinophysistoxin 1 and 2 (DTX 1, 2), yessotoxins (YTX, homoYTX, 45-OH-YTX, 45-OH homo-YTX), azaspriacids (AZA1, 2, 3), pectenotoxins (PTX2, 1, 11), spirolide-1 (SPX1), and gymnodimine (GYM). These were included due to prior reports of YTX-like compounds and activity in *Coolia* [22,23].

For emerging lipophilic toxins (method B), a methanolic 1 mM ammonium fluoride mobile phase was used, along with MS source conditions as described in [61]. Analytes targeted in these analyses included brevetoxins (BTXB2, BTXB4, BTXB5, s-desoxyBTXB2; and PbTX2, PbTX3), pinnatoxins (PnTX A, D, E, F, G, H), additional spirolides (13,19 didesmethyl spirolide C and 20-methyl spirolide C), and an additional gymnodimine (12methyl GYM), which have been associated with sodium channel activity in past studies [61,62]. Individual MRM transitions and parameters are described in Table 4 and were optimized prior to analysis under flow conditions. Data acquisition and analysis were performed using MassLynx and TargetLynx v4.2(Waters, Milford, MA, USA).

Analysis for hydrophilic sodium channel neurotoxins (method C) was conducted using hydrophilic interaction chromatography coupled to tandem mass spectrometry (HILIC-MS/MS) of desalted aqueous fractions based on the method of Turner and colleagues [63]. Following carbon SPE cleanup using Supelclean ENVI-Carb 250 mg/3 mL SPE cartridges and dilution of SPE eluates in acetonitrile (1:3 *v*/*v*), samples were analyzed for tetrodotoxin (TTX) and PSTs including saxitoxin (STX), neosaxitoxin (NEO), the monosulfated gonyautoxins (GTX1, GTX2, GTX3, GTX4, GTX5, GTX6), disulfated STXs (C1, C2, C3, C4), decarbamoylated STXs (dcSTX, dcNEO, dcGTX1, dcGTX2, dcGTX3, dcGTX4), and deoxySTX (doSTX)). Chromatographic separation was performed using an Agilent 1290 Infinity II UHPLC with a 20 µL injection loop. An Agilent 6495B triple quadrupole LC-MS with the iFunnel and Jet Stream technology was utilized as the detector. The analysis was performed using fast polarity switching mode with electrospray ionization. Data acquisition was performed using Agilent MassHunter Acquisition software (Version B.08.00; Stockport, Cheshire, UK), while data processing using Agilent MassHunter Quantitative and Qualitative Analysis software (Version 10.0; Stockport, Cheshire, UK). MS/MS acquisition methods were set-up using the source conditions described by [63] and specific MRM transitions summarized in Table 4. Positive mode (ESI+) transitions were used exclusively for STX, NEO, dcSTX, dcNEO, doSTX, and TTX. Negative mode (ESI-) transitions were used exclusively for GTX1, GTX2, dcGTX2, dcGTX1, and C1 (α-epimers). For the remaining analogs (GTX3, GTX4, GTX5, GTX6, dcGTX3, dcGTX4, C2, C3, and C4), a mix of positive and negative MRMs were used.

Standards for targeted analysis were obtained from a variety of sources and were of a certified concentration where possible. For lipophilic toxins, standards were obtained from the Institute of Biotoxin Metrology, National Research Council Canada (NRCC, Halifax, NS, Canada) as detailed in [63]. For emerging lipophilic toxins, standards were obtained from a variety of sources, including Cawthron Natural Compounds, (CNC, Nelson, New Zealand), CIFGA (Lugo, Spain), and MARBIONC, (Wilmington, DE, USA), as detailed in [59]. Hydrophilic toxin standards were also obtained from NRCC, with the exception of doSTX, dcGTX1, and dcGTX4, which were obtained from CNC and were of an uncertified concentration.

### 5.9. LC-HR-MS Analysis

Liquid chromatography high-resolution MS analyses were performed using an Agilent 1200 LC equipped with a binary pump, temperature-controlled autosampler (10 °C), and temperature-controlled column compartment (40 °C) (Agilent Technologies, Missisauga, ON, Canada) coupled to a Q Exactive HF Orbitrap mass spectrometer (Thermo Fisher Scientific, Waltham, MA, USA) with a heated electrospray ionization probe (HESI-II). Chromatographic separation was on a Kinetex 1.7 μm F5 pentafluorophenyl column (100 × 2.1 mm, Phenomenex, Torrance, CA, USA) using gradient elution with mobile phases composed of 0.1% formic acid in H_2_O (A) and 0.1% formic acid in MeCN (B). The gradient was: 0–18 min, 10–80% B; 18–18.1 min, 80–99% B; 18.1–22 min, 99% B; followed by an 8 min re-equilibration at 10% B, with a flow rate of 0.3 mL/min and an injection volume of 5.0 μL.

Full-scan acquisition was performed with positive and negative polarity switching with a mass range of *m*/*z* 750–1400 to target potential polyethers. A second full scan acquisition with a high-mass-range scan was performed from *m*/*z* 1250 to 3500 to evaluate the presence of high molecular weight MTXs. The spray voltage of the source was +4500 V or −2500 V, with a capillary temperature of 340 °C. The sheath and auxiliary gas were set at 40 and 10 (arbitrary units). The probe heater temperature was set at 150 °C and the S-Lens RF level was set to 100. The mass resolution setting was 120,000 with an automatic gain control (AGC) target of 1 × 10^6^ and a maximum injection time of 100 ms per scan.

Product ion spectra in positive and negative mode with a mass range of *m*/*z* 750–1400 were acquired separately using data-dependent acquisition (DDA) of the top three ions in the full scan spectrum with an isolation window of 1 Da. The resolution setting was 120,000 with an AGC target of 2 × 10^6^ and a maximum injection time of 250 ms with a normalized collision energy of 25 eV in both positive and negative ionization modes.

## Figures and Tables

**Table 1:** Summary of extract N2a-MTT interferences and veratrine enhancing bioactivity in *Coolia palmyrensis*.

Extract Fraction	EtOAc	DCM	H_2_O
Partition	Non-specific cytotoxicity ^1^	None	Non-specific cytotoxicity
10% aq MeOH	None	None	None
60% aq MeOH	None	None	None
90% aq MeOH	Non-specific cytotoxicity	None	Veratrine enhancing activity ^2^

^1^ Non-specific cytotoxicity was attributed to the solvent in this case. ^2^ Veratrine-enhancing activity was detected in 7 of 8 tested extracts in this fraction.

**Table 2:** List of peaks highlighted in data-dependent acquisition in the *Coolia palmyrensi*s media C18-SPE-90% fraction with product ions consistent with gambierones (*m*/*z* 219.1378 or 233.1533) or neutral loss of SO_3_.

RT (min)	[M + H]^+^	[M-H]^−^	Proposed Molecular Formula (Neutral)	RDBE	Loss of 18.0106 (-H_2_O)	Loss of 79.9573 (-SO^3^)	*m*/*z* 219.1378 Product Ion	*m*/*z* 233.1533 Product Ion
4.6	1383.6826 *^†^	1399.6755	C_65_H_108_O_32_	12	No	No	Yes	Yes
5.0	1275.8795	1273.8616	C_65_H_126_O_23_	2	No	No	Yes	Yes
5.3	1111.5933	n/d	C_53_H_90_O_24_	9	Yes	No	Yes	No
6.9	1260.6594 ^†^	1294.6643	C_62_H_105_O_25_NS	11	Yes	Yes	No	No
7.5	899.5010 *	915.4976	C_46_H_76_O_18_	9	Yes	No	Yes	Yes
15.4	1163.8562	1161.8408	C_67_H_118_O_15_	9	No	No	Yes	Yes

* [M + H − H_2_O]^+^, ^†^ [M + H − 2H_2_O]^+^.

**Table 3:** Summary of gambierone and 44-methyl gambierone production by *Coolia* spp.

Species	Strain	Location	No. Isolates	Gambierone	44-Methyl Gambierone	Citation
*C. malayensis*	--	Australia	3	na	+	Murray et al., 2020 [26]
	LM036	Brazil	1	na	−	Tibiricá et al., 2020 [24]
	--	Cook Islands	3	na	−	Murray et al., 2020 [26]
	--	Hong Kong	1	na	−	Murray et al., 2020 [26]
	CAWD175, 154	New Zealand	5	+	+/− (2/5)	Murray et al., 2020 & 2021 [25,26]
*C. tropicalis*	--	Australia	3	na	+/− (2/3)	Murray et al., 2020 [26]
	LM141	Brazil	1	na	+	Tibiricá et al., 2020 [24]
	--	Cook Islands	5	na	+	Murray et al., 2020 [26]
	--	Hong Kong	2	na	+	Murray et al., 2020 [26]
	UTS2	?	1	−	+	Murray et al., 2020 [26]
	UTS3	?	1	−	+	Murray et al., 2020 [26]
*C. palmyrensis*	--	Australia	2	na	−	Murray et al., 2020 [26]
	LM112	Brazil	1	na	−	Tibiricá et al., 2020 [24]
	--	Australia	2	na	−	Murray et al., 2020 [26]
	DISL57	USVI	1	+	trace	This study
*C. canariensis*	--	Hong Kong	2	na	−	Murray et al., 2020 [26]
*C. monotis*	--	Spain	1	na	−	Murray et al., 2020 [26]
*C. santacroce*	LM113	Brazil	1	na	−	Tibiricá et al., 2020 [24]

**Table 4:** Mass list for targeted MRM analyses. See Section 5.8 for more details on targeted analytes.

Method	Toxin	Positive Ions (*m*/*z*)	CE	CV	Negative Ions (*m*/*z*)	CE	CV
A	OA, DTX2	-	-	-	803.5 > 255.1; 803.5 > 113	48; 55	70
A	DTX1	-	-	-	817.5 > 255.1; 817.5 > 113	45; 60	70
A	YTX	-	-	-	570.5 > 467.4; 570.5 > 396.2	30	38
A	homo YTX	-	-	-	577.5 > 474.2; 577.5 > 403.2	30	38
A	45 OH YTX	-	-	-	578.5 > 467.4; 578.5 > 396.2	30	38
A	45 OH homo YTX	-	-	-	585.5 > 474.2; 585.5 > 403.2	30	38
A	AZA1	842.5 > 654.4; 842.5 > 362.3	50	42	-	-	-
A	AZA2	856.6 > 654.4; 856.6 > 362.3	50	42	-	-	-
A	AZA3	828.5 > 658.4; 828.5 > 362.3	50	42	-	-	-
A	PTX1/PTX11	892.5 > 821.5; 892.5 > 213.1	25; 37	32	-	-	-
A	PTX2	876.6 > 823.5; 876.6 > 213.1	25; 38	32	-	-	-
A	SPX1	692.5 > 164.1; 692.5 > 444.3	45; 35	42	-	-	-
A	GYM	508.4 > 136.1; 508.4 > 162.1	38	36	-	-	-
B	PnTx E	784.2 > 164.0; 784.2 > 446.2	55; 45	40	-	-	-
B	PnTx F	766.4 > 164.0; 766.4 > 488.2	55; 45	40	-	-	-
B	PnTx G	694.5 > 164.0; 694.5 > 458.1	55; 50	40	-	-	-
B	BTX B2	1034.5 > 929.0; 1034.5 > 947.0	40	40	-	-	-
B	BTX B4	1272.7 > 929.4; 1272.7 > 326.2	35	80	-	-	-
B	BTX B5	911.5 > 875.5; 911.5 > 839.0	20	60	-	-	-
B	PbTx 2	895.5 > 319.2; 895.5 > 877.5	30	60	-	-	-
B	PbTx 3	897.5 > 725.5; 897.5 > 129.0	30	60	-	-	-
B	S desoxy BTX B2	1018.6 > 248.2; 1018.6 > 204.1	40	40	-	-	-
B	13, 19 didesmethyl spirolide C	678.5 > 164.1; 678.5 > 430.1	50; 35	40	-	-	-
B	20-methyl spirolide C	706.5 > 163.6; 706.5 > 346.2	50; 35	40	-	-	-
B	12methyl GYM	522.7 > 135.0; 522.7 > 120.5	40	40	-	-	-
B	PbTX 1	867.2 > 221.0; 867.2 > 611.0	30	60	-	-	-
B	PnTX A	712.5 > 458.3; 712.5 > 164.1	40	40	-	-	-
B	PnTX D	782.0 > 164.0	55	40	-	-	-
B	PnTX H	708.0 > 164.0	55	40	-	-	-
C	STX	300.1 > 204.1, 138.0	31; 24	-	-	-	-
C	NEO	316.1 > 126.1, 298.1, 220.1	30; 20	-	-	-	-
C	dcSTX	257.1 > 126.1, 222.0	20; 20	-	-	-	-
C	dcNEO	273.1 > 126.1, 225.1	24; 18	-	-	-	-
C	doSTX	241.1 > 60.0, 206.1	25; 20	-	-	-	-
C	TTX	320.1 > 302.1, 162.1	28; 44	-	-	-	-
C	GTX2	-	-	-	394.1 > 351.1; 394.1 > 333.1	20; 18	-
C	GTX3	396.1 > 298.1	18	-	394.1 > 333.1	22	-
C	GTX1	-	-	-	410.1 > 367.1; 410.1 > 349.1	15; 20	-
C	GTX4	412.1 > 314.1	18	-	410.1 > 367.1	15	-
C	GTX5	380.1 > 300.1	15	-	378.1 > 122.0	22	-
C	GTX6	396.1 > 316.1	12	-	394.1 > 122.0	24	-
C	dcGTX2	-	-	-	351.1 > 164.0; 351.1 > 333.1	22; 12	-
C	dcGTX3	353.1 > 255.1	15	-	351.1 > 333.1	18	-
C	dcGTX1	-	-	-	367.1 > 274.1; 367.1 > 349.1	20; 17	-
C	dcGTX4	369.1 > 271.1	20	-	367.1 > 349.1	16	-
C	C1	-	-	-	474.1 > 122.0	38; 30	-
C	C2	396.1 > 298.1	15	-	474.1 > 122.0; 474.1 > 351.1	38	-
C	C3	412.1 > 332.1	12	-	490.1 > 410.1	16	-
C	C4	412.1 > 314.1	12	-	490.1 > 392.1	20	-

## Supplementary Materials

The following supporting information can be downloaded at https://www.mdpi.com/article/10.3390/md21040244/s1: Figure S1: Representative CTX3C curve on N2a-MTT assay, Figure S2: Interference of H_2_O partition controls on the N2a assay prior to SPE; Figure S3: Interference of H_2_O partition controls on the N2a assay following clean-up via flash chromatography with C18-SPE, Figure S4: Interference of EtOAc partition controls on the N2a assay prior to SPE, Figure S5: Interference of EtOAc partition controls on the N2a assay following clean-up via flash chromatography with C18-SPE, Figure S6: Effect of gambierone, 44-methylgambierone, and CTX3C standards on the N2a-MTT assay.
