# Supplementary material for: Gambierone and Sodium Channel Specific Bioactivity Are Associated with the Extracellular Metabolite Pool of the Marine Dinoflagellate Coolia palmyrensis
Source: Mar Drugs. 2023 Apr 15;21(4):244. doi: 10.3390/md21040244 (PMC10143066; doi:10.3390/md21040244)
Supplement: Supplementary file 1 [file marinedrugs-21-00244-s001.zip › marinedrugs-2165874-supplementary.pdf]

Supplementary Materials

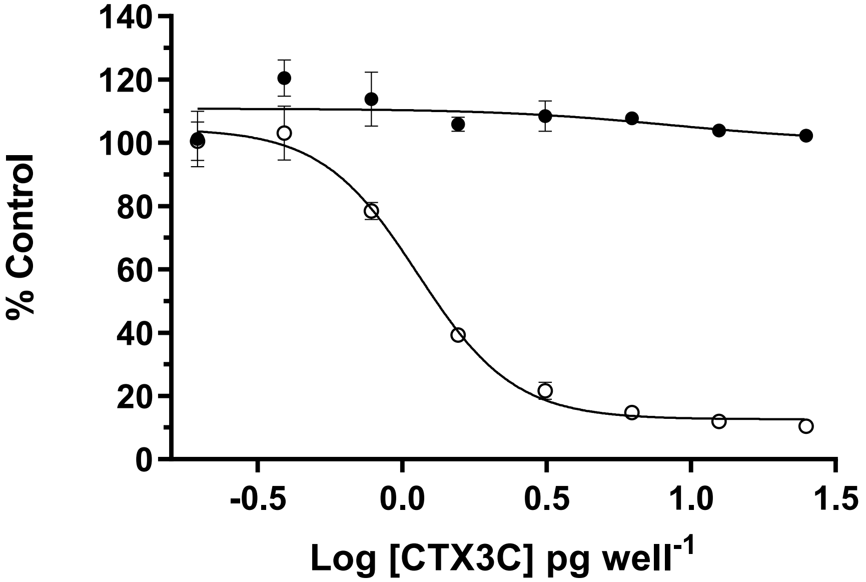

Figure S1. Representative CTX3C curve on N2a-MTT assay. CTX3C curves were run along-side each assay conducted on samples to qualitatively confirm the response of +OV and -OV treatments to CTX3C standard. Serial dilutions of toxin standards were dosed with (+OV; open symbols) and without (−OV; closed symbols) ouabain and veratrine. Data were normalized as a percent to their respective OV controls and each data point represents the mean ± SD. In some cases the error bars are obscured by the symbols. At least two standard curves were performed with each round of assays for QAQC purposes.

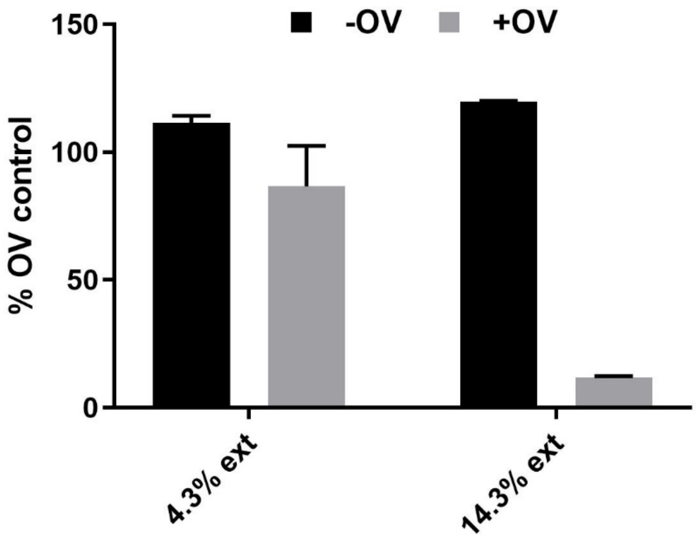

Figure S2. Interference of H_2O partition controls on the N2a assay. Controls consisted of fresh K-media extracted with HP-20 in the same manner as experimental samples. Bars represent mean of triplicate controls ± SE.

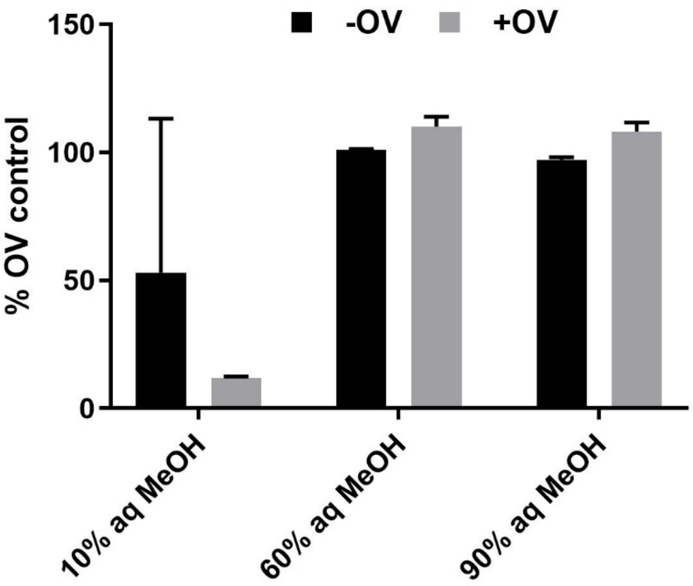

Figure S3. Interference of H_2O partition controls on the N2a assay following clean-up via flash chromatography with C18-SPE. Controls consisted of fresh K-media extracted with HP-20 in the same manner as experimental samples. Bars represent mean of triplicate controls ± SE.

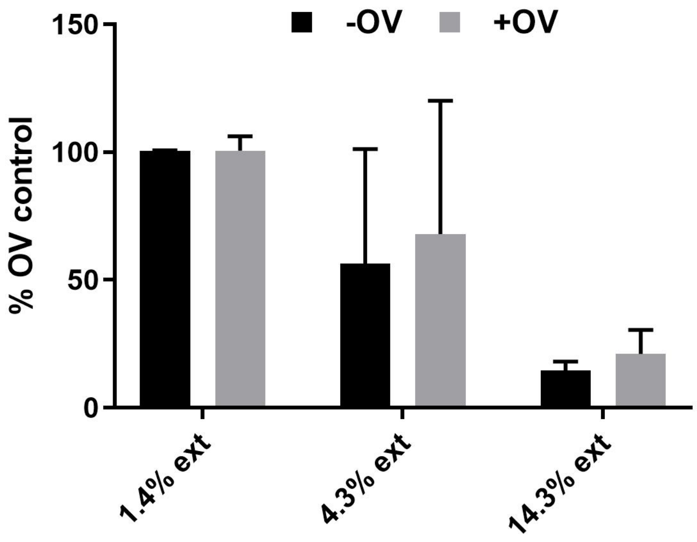

Figure S4. Interference of EtOAc partition controls on the N2a assay. Controls consisted of fresh K-media extracted with HP-20 in the same manner as experimental samples. Bars represent mean of triplicate controls ± SE.

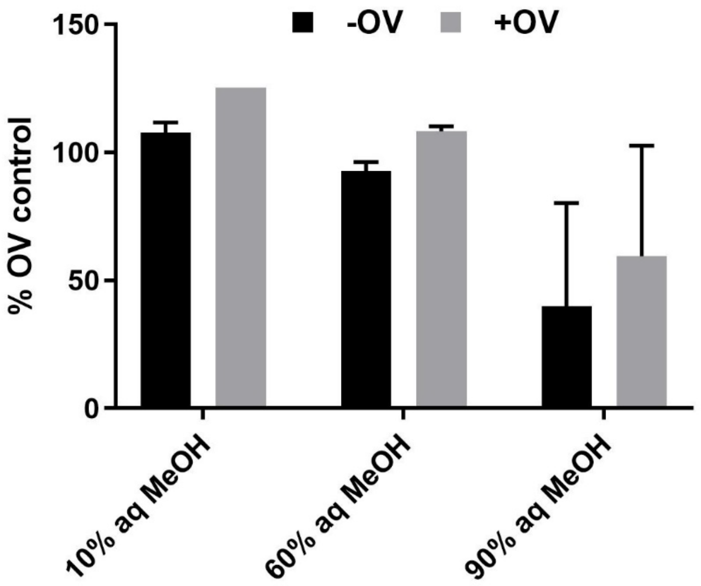

Figure S5. Interference of EtOAc partition controls on the N2a assay following clean-up via flash chromatography with C18-SPE. Controls consisted of fresh K-media extracted with HP-20 in the same manner as experimental samples. Bars represent mean of triplicate controls ± SE.

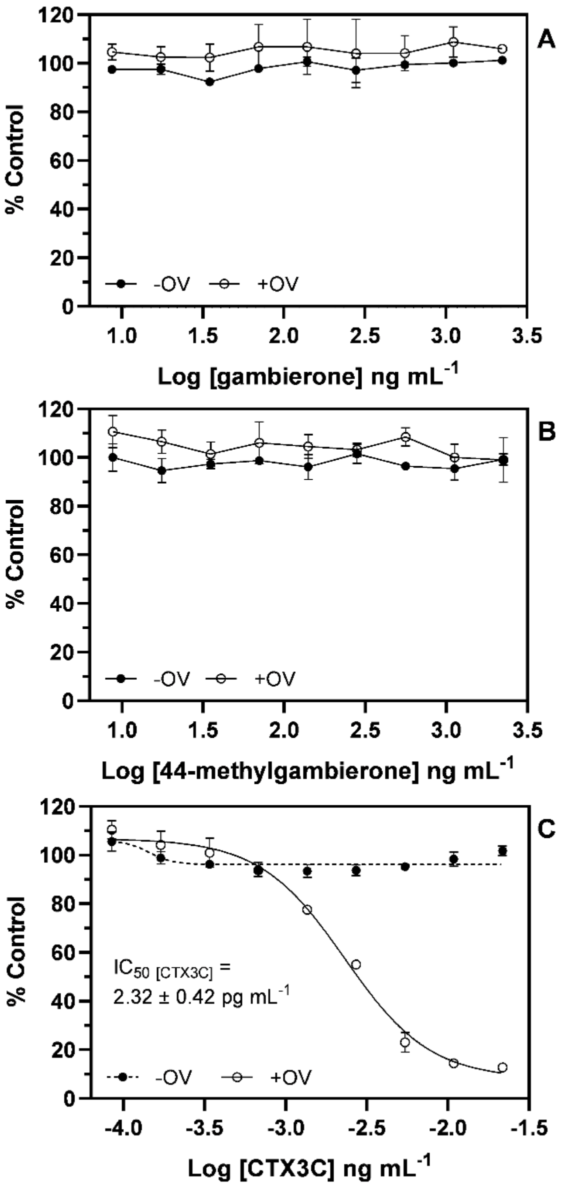

Figure S6. Effect of gambierone (**A**) and 44-Methylgambierone (**B**) on the N2a-MTT assay compared to a CTX3C (**C**). Serial dilutions of toxin standards were dosed with (+OV; open symbols) and without (−OV; closed symbols) ouabain and veratrine. Data were normalized as a percent to their respective OV controls (% control) and obtained from three independent assays, all conducted in triplicate. All concentrations were converted to the same units (log [ng mL^{-1}]) for ease of comparison. The mean ± SD between measurements is plotted and IC_{50} calculated for CTX3C in pg mL^{-1} (text inset).
